# Woodchuck Hepatitis Virus Post-Transcriptional Regulation Element (WPRE) Promotes Anti-CD19 BiTE Expression in Expi293 Cells

**DOI:** 10.52547/ibj.25.4.275

**Published:** 2021-05-19

**Authors:** Reza Moazzami, Hasan Mirzahoseini, Leila Nematollahi, Farzaneh Barkhordari, Mozhgan Raigani, Fatemeh Hajari Taheri, Fereidoun Mahboudi, Fatemeh Davami

**Affiliations:** 1 Biotechnology Research Center, Pasteur Institute of Iran, Tehran, Iran;; 2 Department of Immunology, Hybridoma Lab, Pasteur Institute of Iran, Tehran, Iran

**Keywords:** Acute lymphoblastic leukemia, Bispecific antibodies, Monoclonal antibody

## Abstract

**Background:**

Bispecific antibodies represent an important class of mAbs, with great therapeutic potentials due to their ability to target simultaneously two distinct epitopes. The generation of functional bispecific antibodies with the highest possible yields is particularly critical for the production of these compounds on industrial scales. Anti- CD3 × CD19 bsAb is a bispecific T-cell engager (BiTE) currently used for treating ALL. Herein, we have tried to optimize the expression level of this antibody in mammalian hosts.

**Methods:**

WPRE sequence was incorporated at the 3’ end of the expression cassette. This modification resulted in a notable about two-fold increase in the expression of the bsAb in the Expi293 cell line.

**Results & Conclusion:**

Follow-up flow cytometry analysis demonstrated the binding properties of the produced antibody at acceptable levels, and *in vitro* bioactivity assays showed that this product is potent enough for targeting and destroying CD19-positive cells. Our findings show that WPRE enhances the expression of this type of bispecific mAbs in HEK-293 family cell lines. This approach can be used in biopharma industry for the mass production of anti-CD3 × CD19 bispecific antibody.

## INTRODUCTION

Immunotherapy of cancer is considered as a successful approach in treating a wide spectrum of malignancies, including leukemia and lymphoma^[^^1^^]^. A range of cancer immunotherapy agents have been introduced including mAbs, checkpoint inhibitors, and cancer vaccines. mAbs have a major impact on cancer immunotherapy, as shown by their successful implementation for more than a decade^[^^2^^]^. Presently, there is a broad range of mAbs capable of specifically targeting different cell surface antigens, making them a valuable and reliable tool in this new targeted therapy^[^^3^^]^. Despite their undeniable applications in research, diagnosis, and therapy, native mAbs suffer from some critical limitations viz a restricted activity, Fc-dependent mechanism of action, inadequate pharmacokinetic profile, and limited tissue accessibility^ [^^4^^-^^6^^]^. To evade such limitations, numerous strategies, including engineered antibodies, have been developed in recent years^[^^7^^]^.

Bispecific antibodies are a successful subclass of engineered antibodies, which hold promise as a method for increasing mAb efficacy through the combination of two activities in one molecule. These activities include neutralizing two ligands, inhibiting two receptors, crosslinking two receptors on one cell, and redirecting cytotoxic immune cells^[^^8^^,^^9^^]^. Until now, regulatory authorities have approved three bispecific antibodies, blinatumomab (Amgen, USA), catumaxomab (TriOn Pharma, UK)^[^^10^^]^, and Emicizumab (Roche, Switzerland), for clinical uses^[^^11^^]^. Blinatumomab is the first in the class member of the bispecific T cell-engaging (BiTE®) mAbs, which can direct T cells toward target tumor cells^[^^12^^]^. The simultaneous binding of blinatumomab to cytotoxic CD3^+^ T cells and CD19^+^ B cells results in malignant B cell lysis^[^^13^^]^, while that of the CD19 marker and CD3 causes the T-cell activation, leading to the up-regulation of INF-, TNF-α, CD69, CD25, and CD2, including *ILs-2*, *-6*, and -*10* genes^[^^14^^]^. Additionally, the secretion of granzymes and perforin from cytotoxic T lymphocytes mediates cell lysis^[^^15^^]^, giving rise to polyclonal T-cell activation and proliferation^[^^1^^]^. CD19 is the first B-lineage-specific antigens appearing on the cell surface of B lymphocytes retained in all stages of B-cell development, except in plasma cells^[^^16^^]^. Blinatumomab is applied to treat relapsed or refractory Philadelphia chromosome-negative B-ALL^[^^17^^]^. ALL is a type of lymphoid line malignancies, marked by the rapid dispersion of lymphoblasts in bone marrow^[^^18^^]^. In spite of the great progress recorded in chemotherapy and hematopoietic stem cell transplantation, B-ALL patients continually experience the high rates of relapse, highlighting the need for novel therapies^[^^19^^]^. 

Currently, a variety of bispecific antibodies have been successfully expressed in several hosts, and some of them are in trials. An anti-CD123 × anti-CD3 in BiTE-Fc format was transiently expressed in the CHO-K1 cell line for treating leukemia^[^^20^^]^. Moreover, there are promising results for treating P-cadherin expressing solid tumors by the application of a bsAb targeting P-cadherin and CD3 in DART-Fc format in CHO cell^[^^21^^]^. Recently, Roche has developed a vascular endothelial growth factor-A and angiopoietin-2 targeting bispecific antibodies in CrossMab format for treating age-related macular degeneration. This product, expressed in the HEK293-F system (Invitrogen, USA), is in clinical trial Phase II^[^^22^^]^. Expi293F™ cells were used for the production of bispecific CrossMab that targets CD4 and the HIV envelope glycoprotein^[^^23^^]^. Expi293F™ cells (Gibco, USA) are a suspension derivative of the HEK cells, adapted to grow at high cell densities in a CD, serum-free medium for transient gene expression^[^^21^^]^. In mammalian hosts, the transient gene expression, in contrast to the generation of stable cell lines, ensures the rapid production of enough target proteins for biophysical and biochemical assessment, as well as preclinical studies^[^^24^^-^^26^^]^. Engineering the expression vector, to enhance protein production levels per gene copy, is a prominent approach used in transient gene expression^[^^27^^]^. Importantly, PTREs in mammalian cells can improve gene expression levels through the increased mRNA stability, polyadenylation, translation, and export to cytoplasm^[^^27^^,^^28^^]^. Existing evidence indicates that the presence of WPRE may result in increased expression levels of target proteins through acting on polyadenylation, mRNA export, or translation^[^^28^^-^^30^^]^. Therefore, WPRE can be used to enhance the expression levels of target proteins in transient gene expression. Curiously, some reports have demonstrated the ability of WPRE to elevate the expression level of transgenes in a variety of cell lines^[^^27^^,^^31^^]^.

To date, the impact of WPRE on transgene expression, which is cell line- and construct-dependent, has not yet been investigated in bispecific production and in Expi293 cells. Hence, we aimed at studying the effect of WPRE on the expression level of anti-CD3 × CD19 bsAb (blinatumomab) in the Expi293 cell line.

## MATERIALS AND METHODS

**Cell lines and reagents**

Suspension-adapted Expi293F™ cells was purchased from Invitrogen and NALM-6 and Jurkat cell lines from the National Cell Bank of Iran (Tehran). CD, protein-free Expi293™ Expression Medium, and ExpiFectamine™293 transfection Reagent were procured from Invitrogen. Penicillin/streptomycin and L-glutamine were also obtained from Invitrogen. Ni-NTA Superflow resin was acquired from Qiagen (USA). HRP-conjugated anti-polyhistidine antibody, trypan blue, and DAB were bought from Sigma-Aldrich (USA). Calcein AM viability kit and Saponin were from Trevigen (USA) and Roche (Mannheim, Germany), respectively. Cell separation media were purchased from Cedarlane (Canada), and FCS and RPMI 1640 from Gibco (Karlsruhe, Germany). An anti-polyhistidine PerCP-conjugated antibody was acquired from R&D systems (Minnesota, USA).

**Cell culture conditions**

Expi293F™ cells were cultured in a serum-free and CD Expi293™ Expression Medium supplemented with 2 mM of penicillin/streptomycin. The cells in the culture media were grown in glass bottles on a shaker in a humidified 5-8% CO_2_ incubator at 125 rpm at 37 °C. Transfection was performed using ExpiFectamine™293 reagent according to the manufacturer’s instruction. Every three days, the cells were passaged at a density of 3 × 10^5^ cells/mL, and the cell density and viability ratio were evaluated using trypan blue exclusion methodology. 

**Expression cassettes**

To make the blinatumomab expression constructs, pcDNA3.1 (+) vector (from Invitrogen) was used as the backbone. The synthetic sequences corresponding to the bsAb and WPRE were obtained from a commercial supplier on a single pGH plasmid. For this study, two constructs were prepared: (1) pcDNABi (7 kb) by inserting the bsAb sequence extracted from the pGH plasmid (using *Nh*eI and *Hind*III cut sites) into the destination vector pcDNA3.1, and (2) pcDNABiW (7.5 kb) by inserting "bsAb + WPRE" sequence extracted from the pGH plasmid (using *Nhe*I and *Eco*RI) into pcDNA3.1. 

**Transfection of Expi293F cells**

 Cells were transfected using cationic lipid-based ExpiFectamine™293 Reagent, according to the manufacturer’s protocol. One day before transfection, 2.5 × 10^6 ^cells/mL were seeded onto a six-well plate, followed by the addition of 2 mL of Expi293™ Expression Medium with no supplement. The next day, a transfection cocktail, formed at a ratio of 2.7 µg:1 µL (DNA-ExpiFectamine™293 Reagent), was diluted in 200 µL of serum-free media. After 30 minutes of incubation at room temperature, DNA and ExpiFectamine™293 were added to the cells. In the next step, i.e. 16–18 hours after transfection, 10 µL of ExpiFectamine™ Transfection Enhancer 1, and 100 µL of ExpiFectamine™ Transfection Enhancer 2 were added to each well according to the manufacturer’s protocol. The cells harvested on day seven were centrifuged 200 ×g at room temperature for five minutes, and the supernatant fractions were kept at -20 °C for future analysis. To survey the effect of WPRE on mAb expression in Expi293F™ cells, the GFP-expressing pb513b1 plasmid was used as an internal control vector for the normalization of sample-to-sample transfection efficiency variations using flow cytometry assays after 48 and 72 hours following transfection. For flow cytometry, viable cells were gated and analyzed quantitatively using CyFlow software to evaluate the expression of GFP. 

**Target and effector cell preparation**

CD19-positive B cell (NALM-6) and CD3-positive T cell (Jurkat) lines were cultured in 10% FCS supplemented with complete RPMI 1640. Blood samples were obtained from healthy donor volunteers. Ficoll density gradient centrifugation was used for isolating PBMCs from the buffy coat. Erythrocyte lysis buffer was used for erythrocytes removal (15 minutes at room temperature), followed by centrifugation at 600 ×g for 5 minutes. Lysed erythrocytes containing supernatant was discarded, and the cells were resuspended in PBS. Then the platelets were removed with an extra centrifugation step at 100 ×g for 15 minutes. PBMCs were resuspended in RPMI 1640 to be used the following day without stimulation.

**Protein purification**

Antibody purification was performed by Ni-NTA superflow resin. Filtrated supernatant (using a 0.45-µm filter) was introduced to a 1-mL column at 1 mL/min flow rate. Column equilibration was performed by applying an equilibration buffer containing NaH_2_PO_4_ (50 mM), NaCl (300 mM), and imidazole (10 mM), pH 8.0, and the sample was then loaded on the column. In the next step, a wash buffer, comprising of NaH_2_PO_4_ (50 mM), NaCl (300 mM), and imidazole (20 mM), pH 8.0, was used, and in the end, the column was eluted by an elution buffer consisting of NaH_2_PO_4_ (50 mM), NaCl (300 mM), and imidazole (250 mM), pH 8.0. The bsAb containing fraction was collected in 1.5-mL vials and kept at -20 °C. A Nanodrop spectrophotometer was used to measure the protein concentrations.

**SDS-PAGE analysis and Western blotting**

The expressed mAb was measured by SDS-PAGE and Western blotting. Purified fractions were subjected to 12% polyacrylamide gel (SDS-PAGE) and stained with Coomassie Brilliant Blue. For the Western blot analysis, separated proteins were transferred onto a nitrocellulose membrane using a semidry transfer cell (BioRad, USA). The membrane was blocked in 4% bovine serum albumin at 4 °C overnight, then incubated with HRP-conjugated anti-polyhistidine antibody (1:1000 dilution) for 120 minutes. The bands were then visualized by DAB.

**Flow cytometry analysis**

Binding properties of the expressed bispecific mAb was assessed by flow cytometry (Partec, Germany). For this purpose, 1 × 10^6^ cells (Jurkat, PBMC, NAML-6, or CHO) were mixed with bscCD19 × CD3 (200 µg/mL in PBS) at 4°C (50 µL of final volume) for 30 minutes. Subsequently, the cells were washed twice with FACS buffer (containing PBS and 1% FCS) and centrifuged at 400 ×g at 4 °C for 5 minutes. Cells were then incubated with secondary antibody (anti-His-tag PercP-conjugated antibody) on ice in the dark for 30 minutes, washed twice in the same conditions and analyzed by flow cytometry in the FL3 channel. The experiment was negatively controlled using the His-tag antibody. Flow cytometry output was analyzed by CyFlow (Partec, Germany). 

**Bioactivity assay**

The bsAb cytotoxicity was analyzed using the cytotoxic assay. PBMC preparation was performed as explained above, and T-cell isolation was performed using nylon wool method^[^^32^^]^. About 1.2 × 10^7 ^NALM-6 cells were labeled, in a cell culture medium, with 10 µM of calcein-AM (Tervigen, USA) at 37°C for 30 minutes. After washing two times in the cell culture medium, the cells were adjusted to 3 × 10^5^ cells/ml density in RPMI (10% FCS), and about 30,000 cells were aliquoted in 100-µl volumes for application per reaction assay. Isolated PBMCs were washed with RPMI (10% FCS) and adjusted in the same medium to the desired E/T ratio, and bsAb dilution to the required concentrations was carried out using PBS/0.1% human serum albumin. The cytotoxic reaction was performed by mixing 0.3 × 10^5^ calcein-AM-labeled target cells with 0.3 × 10^6^ T cells or PBMCs (E/T ratio of 10:1) in 5% CO_2 _at 37 °C, followed by the addition of 20 µl of antibody (200 µl of total volume). Cell lysis was then assessed after 4 hours. The released fluorescent dye into the supernatant was quantified by synergy4 fluorescence reader (Germany). The fluorescence signal was measured from cells lacking cytotoxic compound, while the bsAb was used as a negative control. For positive control NALM-6 cells (without bscCD19 × CD3) were nonspecifically lyzed in 1% saponin for 10 minutes (20 µl of final volume). Specific cytotoxicity was calculated based on the following formula:

F(sample)-F(control)/[F(total lysis)-F(control)] × 100

**Ethical statement**

The above-mentioned sampling protocols were approved by the Research Ethics Committee of Pasteur Institute of Tehran, Iran (ethical code: IR.PII.REC.1394.38). Written informed consents were provided by all the participants. 

## RESULTS

**Expression vector construction**

**Fig. 1:**
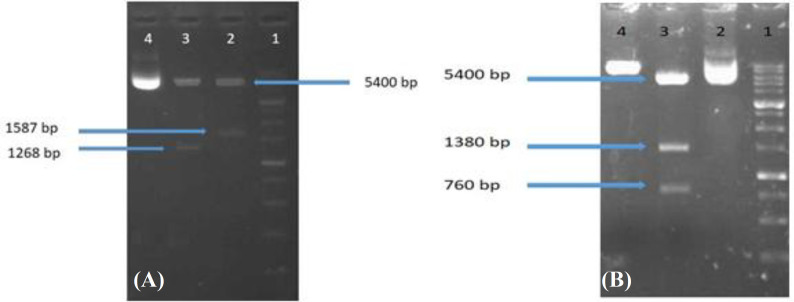
Cloning confirmation of pcDNABi (A) and pcDNABiW (B). (A) Lane 1, 1-kbp DNA marker; lane 2, digested pcDNABi vector by *Nhe*I and *Hind*III restriction enzymes; lane 3, *Xho*I digested pcDNABi vector; lane 4, undigested pcDNABi vector; (B) Lane 1, 1-kbp DNA marker; lane 2, undigested pcDNA3.1 vector; lane 3, digestion of pcDNABiW vector with *Eco*RV generating three fragments: 5400, 1400, and 760 bp; lane 4, digested the pcDNABiW vector with *Nhe*I restriction enzyme

The encoding sequence of blinatumomab (a BiTE antibody) was cloned into the multiple cloning site of pcDNA3.1 (+) vector using *Nhe*I and *Hind*III restriction enzymes to produce the first construct, pcDNABi. Cloning confirmation was performed by double digestion using the two mentioned enzymes, showing the excision of a 1600-bp insert (Fig. 1A). For making the second construct, pcDNABiW, the DNA sequences of antibody and WPRE element were successfully cloned into the pcDNA3.1 (+) vector using *Nhe*I and *Eco*RI restriction enzymes, and then cloning was confirmed by *Eco*RV digestion of the final construct (Fig. 1B). The fidelity of cloning for both constructs was ultimately confirmed by sequencing.

**Expression analysis**

**Fig. 2:**
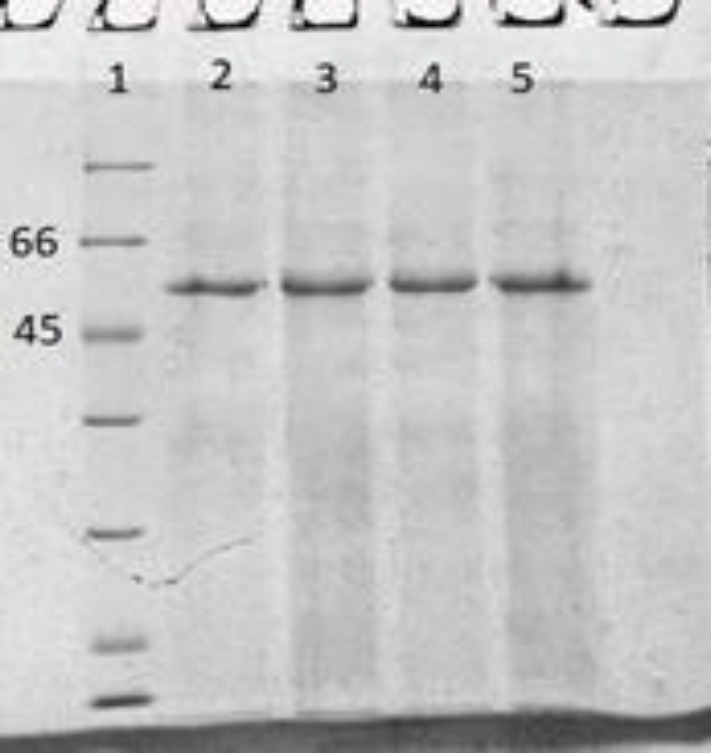
SDS-PAGE analysis of a purified bscCD19 × CD3. Lane 1, protein ladder 116-14 kDa; lanes 2-5, the eluted fraction of bsAb

**Fig. 3:**
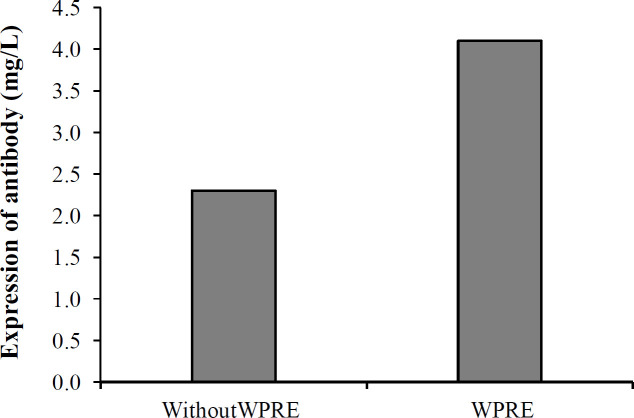
The mAb expression level in Expi293F™ cells in the presence and absence of WPRE element. WPRE enhanced the expression level of bscCD19 × CD3

**Fig. 4:**
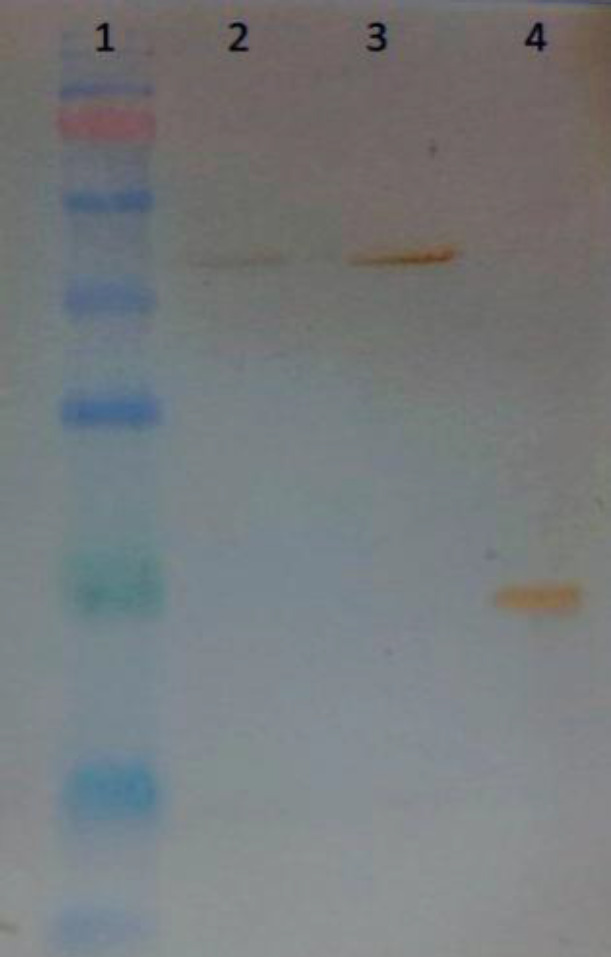
Western blot analysis of bsAb with HRP-conjugated anti-polyhistidine antibody. Lane 1, protein ladder (10–180 kDa); lanes 2 and 3, eluted supernatant of transfected Expi293F™ cells; lane 4, His-tag positive control

 Following transient transfection of Expi293F™ cells, the expressed bsAb was purified from the supernatant of the cells, bearing a yield of 2.3 mg/L. For the transient expression of recombinant antibody in Expi293F™ cells, a mixture of pcDNABi and pb513b1 or a mixture of pcDNABiW and pb513b1 was independently co-transfected into the cells. The pcDNABi or pcDNABiW ratio to pb513b1 was 1:10 in each experiment. On day seven post transfection, the expressed bsAb was collected from the supernatant of each set of transfected cells independently, followed by elution under identical conditions (using the same column, sample volume, and elution condition) for comparison (Fig. 2). The eluted fraction was measured using a NanoDrop spectrophotometer (Thermo Fisher Scientific, USA). Interestingly, the production of bsAb showed a 1.8-fold increase in WPRE plus construct (pcDNABiW) compared with antibodies expressed and purified from WPRE minus construct (pcDNABi). Spike-in control using GFP-expressing reporter plasmid (pb513b1) was applied for the normalization of sample-to-sample variations in transfection. Assessment by flow cytometry showed negligible differences between the two experiments, i.e. transfection of pcDNABi and pb513b1 or transfection of pcDNABiW and pb513b1 (Fig. 3). The eluted protein was detected by anti-polyhistidine antibody conjugated to HRP demonstrated by Western blot analysis. The molecular weight of the purified protein was detected to be around 55 kDa (Fig. 4).

**Binding properties**

**Fig. 5:**
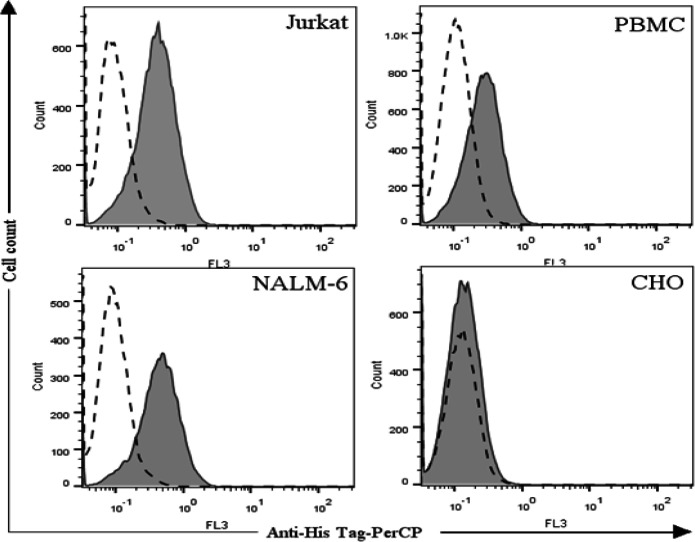
Binding properties of the bscCD19 × CD3. Binding specifications were evaluated by flow cytometry. The Jurkat cell line and PMBCs from healthy donor were used to assess the anti-CD3 binding potency of the bscCD19 × CD3, while CD19-positive B-cell line (NALM-6) were used to evaluate binding properties of the CD19 end of the bsAb. Anti-His-tag PerCP conjugate was employed as a secondary antibody. As shown in the Figure, both ends of bscCD19 × CD3 reveled significant binding to their receptor. CHO (CD3^-^ CD19^-^) cells were applied as a negative control, showing no binding of the bscCD19 × CD3 to this cell. Dotted lines represent cells untreated with bsAb

Binding properties of the expressed bsAb bscCD19 × CD3 was evaluated by flow cytometry. To assess the binding properties of the anti-CD3 moiety, we used two cell types, Jurkat cells as a CD3^+ ^cell line and PBMC (isolated from healthy human peripheral blood containing CD3^+ ^T lymphocytes). Both cell types were incubated with the purified bscCD19 × CD3 antibody (200 µg/mL) and then labeled with the anti-His-tag-PerCP as the secondary antibody (see details in Materials and Methods). As shown in Figure 5, attachment of the bscCD19 × CD3 to the CD3 receptor was observed in both Jurkat CD3^+ ^cell line and PBMC.

The CD19^+^ B-cell line (NALM-6) was used to evaluate the binding properties of the CD19 moiety. For this purpose, NALM-6 cells incubated with the primary bsAb (bscCD19 × CD3) were mixed with anti- His-tag-PerCP, a PerCP-labeled anti-His IgG antibody (as mentioned in Materials and Methods), followed by FACS analysis that showed acceptable binding properties. Interestingly, no binding was detected on the CD19, the CD3 double-negative CHO cell line (Fig. 5).

**Bioactivity assay**

**Fig. 6:**
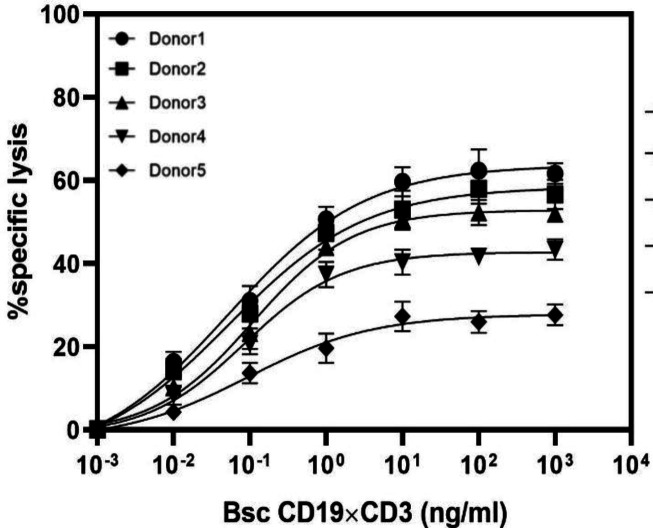
Dose-response analysis of peripheral T cell of five healthy donors (effector cells) and NALM-6 cells (target cells). Calcein-AM release assay was performed at 10:1 ratio after four hours. Error bars give mean (± standard deviation) values of triplicated measurement

**Fig. 7:**
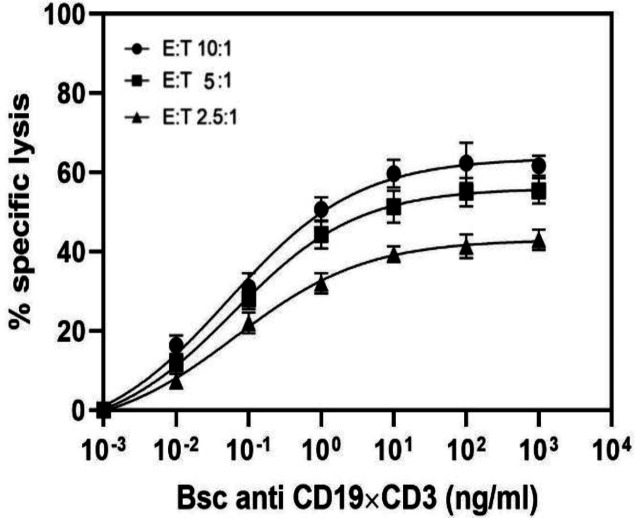
The effect of different E/T ratios on cell lysis activity of bscCD19 × CD3. Dose-response analysis is based on calcein-AM cytotoxicity assay. Error bars give mean (± standard deviation) values of triplicated measurement

The fluorochrome calcein-AM^33^ was used as a fluorescence-based cytotoxicity assay, which is trapped inside living cells and released only upon cell lysis, resulting in the appearance of the fluorescent dye in the supernatant. Calcein-AM-labeled NALM-6 B cells were mixed with T cells isolated from PBMCs at a 10:1 ratio. After four hours of incubation, calcein-AM was determined in the cell culture medium (sup) by a fluorometer. Figure 5 indicates the dose-response curves of five donor T-cell preparations. A variable percentage of cell lysis and different ED_50_ values were obtained with effector cells from five donors. The value and percentage of ED_50_ and cell lysis ranged from 52 pg/ml to 109 pg/ml and 26% to 62%, respectively. Notably, we observed that the ED_50_ value was inversely proportional to the cell lysis, i.e. the higher the percentage of cell lysis, the lower the ED value. However, the correlation was not completely linear (Fig. 6). In four out of the five cases, bsAb was effective in concentrations below 100 pg/ml, and the mean of ED_50_ value for these five donors was 78 pg/ml (Fig. 6). To measure the targeting efficiency of bsAb at different E/T ratios, the mAb was examined at several E/T ratios (10:1, 5:1, and 2.5:1) by using NALM-6 and T cells as target and effector cells, respectively. As depicted in Figure 7, the ED_50_ values for these ratios were 52, 61, and 65 pg/ml, respectively. This observation demonstrated that the ED_50_ values slightly increased in response to the decrease in E/T ratios with close cell lysis ratio values of 10:1 and 5:1 (63% vs. 56%, respectively). Our findings proved the effectiveness of anti CD3 × CD19 bsAb at low E/T ratios. Moreover, in contrast to some other CD19-targeting bispecific antibodies, a low E/T ratio is enough for cell lysis.

## DISCUSSION

Mammalian expression systems are important, particularly in the production of biopharmaceuticals. In this context, transient gene expression is a primary step that can cost-effectively produce quite enough amounts of target proteins in a short time for preclinical studies. HEK293 cell line has been used for decades as a cell line of choice for transient gene expression, due to its high transfection efficiency as well as high protein production yield. However, this cell line is adherent, limiting its scale-up and expansion. In contrast, Expi293, a derivative of HEK293, is a suspension cell line with the capability of reaching high cell densities. 

Vector engineering, the same as cell line engineering and process development, is a fundamental approach for enhancing the production yield of recombinant proteins. PTREs have been frequently used to engineer expression vectors, among which WPRE insertion considerably increases the expression level of transgenes in some hosts^[^^27^^, ^^34^^]^.

In the current study, we expressed and purified blinatumomab and measured its cytotoxicity and binding properties in a mammalian expression system and evaluated the effect of the WPRE sequence on the expression levels of the mAb. The bsAb was expressed from a pcDNA3.1 expression vector under the strong Cytomegalovirus promoter activity. Our analysis showed about two-fold increase in the expression level of bispecific antibody when WPRE was present (4.1 mg/L from pcDNABiW vector) compared with the one lacking it (2.3 mg/L from pcDNABi vector). This result is consistent with previous studies that demonstrated WPRE can enhance the expression levels of recombinant proteins. Kim *et al.*^[^^31^^]^ reported that WPRE increases the expression levels of a full-length antibody from 19.3 mg/L to 40.5 mg/L, bringing the enhancement to about 2.1-fold in HEK293E cells. Ho *et al.*^[^^27^^]^ showed WPRE can promote the expression of luciferase by 2.6-fold in HEK293 cells, but had no positive effects in CHO K1 cells. Hlavaty *et al.*^[^^35^^]^ also reported that WPRE enhanced recombinant protein expression level up to three folds in HEK293 cell line, and a combination of WPRE with other PTREs has been shown to increase the expression levels up to 10.5 folds in HEK cells. Besides these positive results, there are also some reports showing no significant enhancements in the expression levels of transgenes by WPRE^[^^27^^]^. This discrepancy can be interpreted as context-dependent effects^[^^36^^]^ or promoter and cell line-specificity and activity^[^^37^^]^ of WPRE. Therefore, particularly for industrial applications, it is important to evaluate the impact of WPRE insertion individually on each specific construct, candidate gene, or host cell line. 

In the present investigation, we demonstrated that the use of WPRE in the pcDNA3.1 expression vector for the expression of bscCD19 × CD3 antibody in Expi293F™ cells effectively increase the expression levels of the transgene. Previous studies have exhibited that bscCD19 × CD3 is effective at low pg/ml ranges and low E/T ratios^[^^38^^,^^39^^]^. Consistent with those reports, functional analysis displayed that the produced antibody using our expression system was capable of lysing target cells at sub ng/ml concentrations and low E/T ratios. In contrast with other bispecific technologies, such as quadroma or diabody requiring much higher concentrations (i.e. 100 nanograms to micrograms of antibodies), the BiTE system induces cell lysis at sub ng/ml concentrations, in a short period of time at low E/T ratios^[^^40^^]^. Additionally, using the T-cell activation of BiTE system is self-sufficient and independent of extra stimuli such as IL-2 and gamma interferon, a requirement for other similar systems^[^^38^^]^.

In this study, we demonstrated the successful production and purification of a bsAb; bscCD19 × CD3, in Expi293 suspension cells via the insertion of a WPRE sequence in the expression cassette. The produced antibody showed full functionality at pg/ml concentrations. Our new expression system could be proposed for the production of this antibody at an industrial scale. 
